# Sex Difference in Cisplatin-Induced Nephrotoxicity: Laboratory and Clinical Findings

**DOI:** 10.1155/2022/3507721

**Published:** 2022-10-10

**Authors:** Fatemeh Eshraghi-Jazi, Mehdi Nematbakhsh

**Affiliations:** Water and Electrolytes Research Center, Isfahan University of Medical Sciences, Isfahan, Iran

## Abstract

Cisplatin (CP) as the most important anticancer drug has limited usage due to a lot of side effects such as nephrotoxicity. Additionally, nephrotoxicity is gender/sex-related. There is a variety of experimental studies in association with sex and CP-induced nephrotoxicity. Some studies have reported that female sex is resistant than male sex due to greater antioxidant defense and protective effects of estrogen in females. Other studies have indicated that males are less vulnerable than females due to CP high clearance. Also, various supplementations have revealed conflicting effects in males and females. It is uncovered that sex hormones have determinant roles on the conflicting effects. Some supplements could improve CP-induced nephrotoxicity, but several supplements intensified CP-induced nephrotoxicity, especially in female sex. On the other hand, major clinical studies introduced female gender as a risk factor of CP-induced nephrotoxicity. Although, rare studies evaluated the effect of various supplemental compounds on CP-induced nephrotoxicity in patients underwent CP therapy. Therefore, it requires further investigations to clarify the controversial subject of gender/sex and CP-induced nephrotoxicity in both clinic and laboratory.

## 1. Introduction

Chemotherapy is one of the therapeutic methods in cancer types, and cisplatin (CP) as an anticancer drug either alone or along with other chemotherapeutic drugs is widely used for treating solid tumors. Although, clinical and experimental studies reported that CP therapy disturbs the function of various organs such as brain [1], ear, kidney, etc. [2,3]. Nephrotoxicity is well known as the most common and important side effect of CP [4], and researchers are attempting to ameliorate or prevent nephrotoxicity in patients subjected to chemotherapy [5]. Recently, gender/sex difference is subjected as a challenging topic, and it has attracted basic researchers [6]. Many experimental studies have reported a variety of findings in association with CP-induced nephrotoxicity and protectant strategies in two male and female genders [7–10]. However, gender difference has been less addressed in the clinical trials. As a result, this issue has been neglected in the therapy process of patients under chemotherapy. This article has reviewed and discussed the effect of gender/sex difference on CP-induced nephrotoxicity in both experimental and clinical studies, separately. Then, it investigated the interaction of supplementations and gender/sex difference on CP-induced nephrotoxicity in both laboratory and clinic fields. Finally, this review has collated experimental and clinical evidences in the association between gender/sex difference and CP-induced nephrotoxicity to answer following questions: Are the mechanisms involved in CP-induced nephrotoxicity different in both sexes/genders? Whether these issues considered in the treatment strategies?

## 2. Search Strategies

A computerized literature exploration comprehensively detected research articles in both experimental and clinical research studies. The keywords including sex, gender, CP, renal toxicity, kidney toxicity, nephrotoxicity, supplement, and treat were searched in the databases of Web of Sciences, PubMed, PMC, Google Scholar, and Scopus without time and language restrictions. In order to combine the keywords, the search operators such as AND, OR, *∗*, and ( ) were applied in the search line.

## 3. Sex Difference in CP-Induced Nephrotoxicity in Laboratory Experiments

Various reports in association between sex difference and CP-induced nephrotoxicity in experimental investigations are available in the literature [11–15]. CP (1 mg/kg/day; for a period of 15 days) increased the markers such as blood urea nitrogen (BUN), creatinine (Cr), magnesium (Mg), and kidney malondialdehyde (MDA) as well as kidney tissue damage significantly in males more than females [11]. Also, Pinches et al. showed that the administration of various doses of CP (0, 0.1, 1 and 2.5 mg/kg) in the time points of 5, 8 and 22 days induced more sensitivity and higher histological lesions in male rats than females, where the changes were notable in the dose of 2.5 mg/kg [15]. Female and male rats receiving either continuous or single dose of CP (2.5  mg/kg) revealed the high 24 h urinary excretion of sodium in male rats, but not in female ones [12]. Also, the single dose of CP induced greater kidney tissue damage in males than that in females, while such observation was not detected in animals treated with the continuous dose of CP [7]. It appears that the single dose administration of CP transport into kidneys high CP amounts all at once, and it makes the deposit of great platinum amount into glomeruli and damages nephrons resulting in the low excretion of CP and sever kidney damage in males. Further, another study found that male mice treated with CP exhibit more severe kidney injury than female mice in both models of chronic kidney disease (CKD) and acute kidney injury (AKI) [16]. In addition, the administration of CP (15 mg/kg) resulted in severe renal injury in wild type male mice than female ones. Although male sex had the low platinum concentration [17] due to the high clearance of CP [18]. All above mentioned studies have declared that female animals are less vulnerable than males ones, and males exhibited greater nephrotoxicity than females. Oxidative stress is one of the mechanisms involving in CP-induced nephrotoxicity. There is a relationship between sex difference and oxidative stress. A literature review illustrated that females have lower oxidative stress and greater antioxidant potential than males [19]. An evidence showed that female mice represented greater resistance than male mice against CP-induced toxicity due to stronger antioxidant system in female than male [9]. In addition, liver and membrane-bound enzymes may participate in gender difference-related nephrotoxicity. An evidence reported that the lower resistance of male mice against CP could be related to the higher expression of liver enzymes involved in CP metabolism than female mice [17]. Also, the participatory role of NADPH-cytochrome P450 reductase (CPR) evidenced that the administration of CP increased the serum BUN level and BUN/Cr ratio accompanied with renal histopathological alterations in male CPR-low mice, but not in female ones [20]. Beside, rat organic cation transporter 2 (rOCT2) as the mediator of CP uptake into renal tubular cells has the higher expression levels of mRNA and protein in male kidney than female kidney [18, 21]. Wild et al. showed that the post-translational regulation of rOCT2 in rat kidney was gender-dependent, and Calmodulin, which was associated with the post-translational regulation of rOCT2, was highly expressed in male rats [22]. Sex steroid hormones also are well known as the most main factor involving in sex difference. It is known that endogenous estrogen hormone has a protective role in females, and it decreases the prevalence of cardiovascular diseases in females [23]. Also, sex hormones affect the expression of rOCT2. Castration decreased the expression level of rOCT2 in male rats [10], and the administration of testosterone enhances the expression of rOCT2 in both male and female genders, while estradiol treatment reduces the expression levels of rOCT2 [24]. In contrast to above mentioned studies, some experiments reported that female sex is more sensitive than male sex. Jilanchi et al. showed that the administration of CP either single (7.5 mg/kg) or continuous dose (3 mg/kg/day; for 5 days) increased the serum levels of BUN and Cr in female more than in male, but neither single nor continuous dose of CP could not induce the sex difference in the increasing of kidney tissue damage [13]. These findings disagreed with Nematbakhsh observations. The study by Nematbakhsh reported that males receiving CP single dose had higher kidney tissue damage than females receiving the same protocol, whereas neither single dose nor continuous dose of CP exhibited no gender difference in the amounts of BUN and Cr [7]. Here, there are two problems. First, why did two protocols of CP administration in Jilanchi^,^s study show similar observations, but not in Nematbakhsh's study? Second, why did females have more renal failure than males in Jilanchi's study, but it was inversed in Nematbakhsh's study? The most main reason of this contrast is the period of study. The periods were 8- and 6- day in Jilanchi and Nematbakhsh's studies, respectively. It seems that the 8-day period is the adequate time for the cumulative dose effect of CP continuous administration. One study reported that female mice represented more nephrotoxicity and higher mortality against CP than male mice, regardless of strain [25]. In addition, a study on male and female monkeys receiving intravenous CP (2.5 mg/kg) illustrated that the BUN and Cr levels had more elevating trends in female than male [26]. A study displayed that the variations of platinum-DNA adduct levels were similar in male and female kidneys until the seventh day of CP post-injection. Although female animals could not survive until the 14th day of CP post-injection [27]. Female sex hormones may be thought to play a protective role in CP-induced nephrotoxicity, but either low or high dose of estrogen may be inefficient or even harmful. It seems that the various levels of estrogen in the different phases of estrous cycle of female animals impress its own protective effects. An evidence indicated that the estrous cycle may affect the activities of hepatic drug-metabolizing enzymes [28]. Additionally, the administration of estradiol could promote CP-induced nephrotoxicity in ovariectomized animals [29]. In addition, hormone therapy in two genders is not able to decline nephrotoxicity induced by both protocols of single and continuous doses of CP [30]. It is reported that both progesterone alone and the combination of estrogen/progesterone could decline CP-induced nephrotoxicity in ovariectomized rats, dose-dependently [31]. It is suggested that progesterone administration is accompanied with estrogen therapy to ameliorate the harmful effects of estrogen alone. In confirming the supposition, progesterone had inhibitory effects on estrogen-induced carcinogenesis in ovariectomized rats [32]. On the other hand, the effects of testosterone also are dose-related, and the administration of low dose testosterone along with CP was able to decline renal tissue damage as well as the serum levels of biochemical indicators [33]. In line with the observation, the antioxidant effects of testosterone administration found in ovareictomized female rats [34].

In overall, the sex difference plays an important role in the development of CP-induced nephrotoxicity, and literature has documented the variety of findings on animal models with various experimental protocols. In laboratory, both male and female sexes can be introduced as risk factors for nephrotoxicity induced by CP. Here, there is a notable point. Age is able to affect gender difference. The administration of CP, as a single dose, could result in renal dysfunction in old male rats than young ones, but not in female rats [35]. In addition, young female mice had the lowest level of plasma Cr, whereas aged female mice exhibited the early and high rate of mortality against kidney acute injury induced by CP [10]. It was evidenced that normal aging gradually decreases reserve capacity in body organs such as kidneys [36]. In addition, the decreasing of sex hormone levels during aging influences kidney function in both genders [37]. Therefore, it is necessary that age also besides sex difference is targeted for further investigations.

## 4. Gender Difference in CP-Induced Nephrotoxicity in Clinical Subjects

Some clinical studies evaluated gender-associated renal toxicity in cancerous patients under chemotherapy with CP, and the studies showed that gender difference was not a predictor for nephrotoxicity induced by CP [3,38–44]. Moreover, CP pharmacokinetics had no association with age, sex, and the measures of kidney dysfunction in patients under chemotherapy [45]. In line with mentioned studies, a prospective hospital-based study reported no gender difference in gentamicin-induced nephrotoxicity [46]. On the other hand, several studies have reported that female gender undergoing chemotherapy is considered as a risk factor in the prevalence of CP-induced nephrotoxicity [2,47,48]. Interestingly, De Jongh et al. showed that women had twice the risk for CP-induced nephrotoxicity than men [2]. Also, it was pointed out a decrement in estimated glomerular filtration rate (eGFR) in women receiving CP over time probably due to low muscle mass [49]. In addition, premenopausal women have presented a greater risk against the progression of CP-induced nephrotoxicity than aged matched men [47]. A retrospective study reported that AKI was more common in female and African American race patients receiving at least 2 cycles of CP high dose [50]. In addition to age and sex, race also is able to propose as one of the risk factors of CP-induced nephrotoxicity. One study showed that ethnicity, sex, and age affect the expression of OCT2 [51]. Niho et al. by a multivariate analysis detected that female gender and age of 71 years or older had a shorter interval between the start of chemotherapy and the development of serum Cr elevation (known as time to serum Cr elevation) at grade 1 or worse than male gender and age below 70 years old [52]. In addition, female gender was reported as an independent risk factor in CP-induced nephrotoxicity in patients under chemotherapy accompanied with short hydration and Mg supplementation [48]. Another evidence showed that short hydration with KCL and MgSO_4_ decreased CP-induced nephrotoxicity among study population under CP therapy; However, a few female patients developed nephrotoxicity [53]. It should be noted that the levels of OCT2 are higher in males than females [54]. In addition, CP pharmacokinetics revealed that platinum clearance is higher in men than women [55], resulted in decreasing CP-induced nephrotoxicity in men. On the other hand, it appears that the delayed removal of CP followed higher nephrotoxicity in women than men. Concerning mentioned clinical studies, apparently, there is a gap. They did not focus on some problems such as oxidative stress and antioxidant defense. One study performed on healthy men and women has reported that women in age groups 35–45 and 46–55 years old had an increasing in the levels of oxidative stress, but it was constant in all age groups in men [56]. In the contrast to mentioned studies, a few clinical studies have reported that men have higher sensitivity against CP-induced nephrotoxicity than women. A comparison between innovator and generic CP formulations in patients under chemotherapy has revealed that the administration of generic CP formulation induced the slight renal toxicity than innovator CP formulation, especially in men [57]. Also, one study designed based on a spontaneous reporting system database, the Japanese Adverse Drug Event Report Database, exhibited that CP-induced AKI occurred in male, hypertensive, and diabetic cancer patients [58]. These findings were not expected because the major clinical studies have expressed that female gender is a risk factor for CP-induced nephrotoxicity in patients under chemotherapy. It is very hard to clarify reasons resulted in these events, and the further evaluations may require in clinical trials. Although the authors have presented some justifications and limitations resulted in the present results. The study by Sekine [57] explained some possibilities: (i) Men had increasing serum Cr due to large muscle mass resulted in producing high Cr. (ii) Man patients should receive more volume of hydration due to large muscle mass than women ones. (iii). Magnesium supplementation was not inserted into hydration fluids, whereas the supplementation could reduce renal toxicity induced by CP [59]. (iv) Mannitol had better administer before CP infusion. (v) Generic and innovator formulations of CP were randomly not assigned in this study. Additionally, Uchida's study [58] designed based on a spontaneous reporting system in Japan informed several topics as following: The data of study were collected by a voluntary reporting system, and there are possible errors in the system. Therefore, the actual number and the incidence rate of AKI are not clear and accurate. Moreover, there was no access to details related to patients such as drug information, patient conditions and clinical data. In spite of these problems, both of Uchida and Sekine's studies could indicate some clinical gaps associated with gender difference, especially in male gender.

Accordingly, women are arguably known as the risk factor of nephrotoxicity induced by CP in the chemotherapy regimen. On the other hand, rare clinical studies reported that men may partly be vulnerable against CP in chemotherapy regimens; however, more evaluations must be designed and done.

### 4.1. The Effect of Supplements on Sex Difference-Related CP-Induced Nephrotoxicity in Experimental Animal Models

The variety of supplemental compounds can be used to ameliorate or prevent CP-induced nephrotoxicity in male and female experimental models, from sex hormones to antioxidants, plant extracts, etc. According to target effects in the review literature, the supplementations are classified as following:

#### 4.1.1. Supplementations Related to the Renin Angiotensin System(RAS)

Losartan, as an angiotensin II type 1 receptor (AT1R) antagonist, could ameliorate CP-induced nephrotoxicity in male rats, but it progressed renal damage in female ones [60]. Losartan also has been known as an antioxidant [61], and its effect on CP-induced nephrotoxicity may be related to its antioxidant effect [62]. On the contrary, enalapril, as the angiotensin I converting enzyme inhibitor, was unsuccessful to decline CP-induced nephrotoxicity in both male and female sexes, even it intensified kidney dysfunction and failure in female sex [63]. Pezeshki et al. revealed that the administration of angiotensin 1–7 could ameliorate renal injury and histopathological changes induced by CP (7.5 mg/kg) in male rats, but not in female rats [64]. Ang 1–7, enalapril, and losartan as vasodilators increase renal blood flow [65,66], considering the higher clearance of CP in males than females [18]. It is suggested that the administration of enalapril, Ang 1–7 or losartan could increase the clearance of CP in males resulted in preventing more renal damage in males. In contrast, increasing RBF in females intensified CP-induced nephrotoxicity due to the lower clearance of CP than males [18].

#### 4.1.2. Supplementations Related to the Nitric Oxide System

Nitric oxide (NO) as a mediator has a critical role in renal hemodynamic [67]. Either inhibition or stimulation of NO follows gender-related responses. The single dose of L-arginine as a NO donor could recover CP-induced nephrotoxicity in male rats, but it exaggerated renal failure in female rats [68]. In addition, the concurrent administration of L-arginine with CP, also recovered CP-induced nephrotoxicity in male rats, but not in female ones [69]. NO acts as a vasodilator agent increases RBF [67]; subsequently, the enhancing clearance of CP attenuates CP-induced nephrotoxicity in males. In confirming the observations, the inhibition of NO synthase by L-NAME not only failed to attenuate nephrotoxicity induced by CP in both male and female sexes but also renal disturbance intensified in male sex [70]. L-NAME is a nonselective NO synthase enzyme inhibitor which inhibits all types of NO synthase enzymes, especially the endothelial NO synthase enzyme resulted in exaggerating endothelial dysfunction. Moreover, the inhibition of inducible NO synthase by S-methylisothiourea hemisulfate (SMT) was able to improve CP-induced nephrotoxicity in male sex than female one, effectively [71]. CP administration induces NO production derived by inducible NO synthase enzyme which participates in the pathogenesis of CP-induced nephrotoxicity [72]. Therefore, the inhibition of inducible NO synthase by SMT involves in ameliorating nephrotoxicity in males, although the subject requires more evaluations. It is suggested to propose a massive project for evaluating the activities of all NO synthase enzymes and their expressions in both male and female genders using selective and nonselective NO synthase enzyme inhibitors as well as applying NO donor agents against CP-induced nephrotoxicity.

#### 4.1.3. Supplementations Related to the Antioxidant System

Some agents such as riboflavin had a stronger ameliorative effect against CP-induced nephrotoxicity in female sex than male one [9]. On the other hand, the intraperitoneal administration of N-acetylcysteine at the doses of 300 and 600 mg/kg failed to eliminate CP-induced nephrotoxicity in both male and female sexes [73]. In another study, animals which underwent renal ischemia reperfusion received NAC at the doses of 150 and 500 mg/kg, and only the low dose of NAC could attenuate both renal dysfunction and lung injury induced by renal ischemia reperfusion [74]. It seems that the effect of NAC is dose-related. An evidence reported that the high dose of some antioxidants not only have not positive effects but also increases mortality [75]. Therefore, in Rajabi's study, both NAC doses are very high that it could not prevent CP-induced nephrotoxicity. In addition, the pomegranate flower extract at the doses of 25 and 50 mg/kg/day for 9 days could not attenuate CP-induced nephrotoxicity in female rats receiving 2.5 mg CP/kg/day for 6 days [76]. El-Arabey commented that the pomegranate flower extract contains phytoestrogen which has estrogenic activity [77]. In a similar study, fennel essential oil did not exhibit protective effects against CP-induced nephrotoxicity in ovareictomized female rats [78]. It is believed that fennel essential oil also contains compounds with estrogenic activity [79]. Moreover, the presence of estradiol also could abolish the renoprotective effects of erythropoietin [80], vitamin E, vitamin C, and losartan [8] in CP-induced nephrotoxicity in ovariectomized rats. It seems that estrogen and compounds with estrogenic activity may have a harmful effect against CP-induced nephrotoxicity in female rats. On the other hand, vitamin E ameliorated CP-induced nephrotoxicity in male rats, but CP-induced nephrotoxicity intensified in female sex [81]. Also, vitamin E may reduce the increasing of the serum level of nitrite induced by CP in males, but not in females [82]. It is appears that vitamin E could improve the endothelial function in males resulted in attenuating CP-induced nephrotoxicity. In contrast, vitamin E had no protective effect in endothelial dysfunction and subsequently in nephrotoxicity induced by CP in female rats. Additionally, erythropoietin was able to recover CP-induced nephrotoxicity in male rats, but it intensified CP-induced nephrotoxicity in females [83]. It was found that the level of endogenous erythropoietin is higher in male than female [84]. Therefore, the administration of exogenous erythropoietin could increase the improvement of CP-induced nephrotoxicity in male sex.

#### 4.1.4. Supplementations Related to Inhibitory Agents

Gamma-aminobutyric Acid (GABA), as an inhibitory neurotransmitter, was unsuccessful to decline CP-induced nephrotoxicity in both male and female rats, even it intensified kidney dysfunction and failure in female ones [85]. GABA has relaxatory effects [86] and could decrease renal vascular resistance [87], probably resulted in increasing renal blood flow and entering more CP amounts to the kidney. Thus, CP-induced nephrotoxicity increased in female sex probably due to lower clearance of CP than male sex. The administration of GABA either as prophylaxis or treatment disturbed the renoprotective effect of hyperglycemia against CP in male, but not in female [88]. It seems that GABA could attenuate the level of glucose in diabetic male rats which lead to eliminate the renal protectant effect induced by high glucose. Maybe, this possibility is unacceptable because this study did not report the level of glucose in the ending of experiment. Also, bosentan, endothelin-1 antagonist, not only was not able to attenuate CP- induced nephrotoxicity in both male and female rats but also it worsened kidney dysfunction and failure in female animals [89]. Bosentan also blocks endotheline-1 receptor and subsequently induces vascular vasodilation. In addition, the plasma level of endothelin-1 is lower in female than male [90], and the administration of estrogen also could decrease the plasma level of endothelin-1 in postmenopausal females [91]. Therefore, it is thought there is a correlation between increasing CP-induced nephrotoxicity and the low plasma level of endothelin-1 in females.

### 4.2. Other Supplements

Karimi et al. showed that dextrose 2% and 10% could not improve CP-induced renal failure and tissue damage in both sexes of male and female, even dextrose 10% exaggerated CP-induced nephrotoxicity in female rats [92]. In addition, female sex is recognized as a risk factor in contrast-induced nephropathy in subjects receiving dextrose [93]. In line with these studies, hydration with mannitol or dextrose also progressed CP-induced nephrotoxicity with higher intensity in females than males [94]. Although hydration with mannitol could be have protective effects on CP-induced nephrotoxicity [95]. An evidence showed that mannitol induced renal vasodilation and increased renal blood flow [96]. Therefore, because females have lower CP clearance than males [18], the increasing renal blood flow may transport more CP into kidney resulted in intensifying nephrotoxicity. As another study, the effect of aerobic exercise against CP-induced nephrotoxicity also was considered which provided unsatisfied results. It reported that aerobic exercise for 8 weeks as prophylaxis could not ameliorate CP (2.5 mg/kg/day for one week)-induced nephrotoxicity in female rats [97]. During exercise, the blood flow increases in exercising skeletal muscles, while blood flow decreases in kidneys [98]. Therefore, because of the decreasing of renal blood flow as well as low CP clearance in females, treadmill exercise could not attenuate CP-induced nephrotoxicity in female sex.

### 4.3. The Effect of Supplements on Gender Difference-Related CP-Induced Nephrotoxicity in Clinical Subjects

A few clinical studies evaluated gender-related renoprotective effects of supplementations in CP-induced nephrotoxicity. It was exhibited male gender as risk factor of AKI in patients underwent chemotherapy and receiving amifostine for reducing nephrotoxicity [99]. On the other hand, either the oral administration of theophylline or the intravenous administration of aminophylline could not ameliorate CP-induced nephrotoxicity in patients receiving chemotherapy, and it was also observed no significant relation between CP-induced nephrotoxicity with age, gender and the dose of CP [100]. One study performed on male mice has demonstrated that amifostine has time-dependent protective effects due to circadian rhythm [101]. Therefore, it had better that protectors have not administered in any day time.

## 5. Clinical Study Limitations

There are some limitations in clinical studies, especially in the gender difference subject. In general, chemotherapy regimens insist of several anticancer drugs. Therefore, the prevalence of nephrotoxicity during chemotherapy is not associated to CP alone. In this regard, a document summarized several points as following: I. The 57.3% of prescription drugs were nephrotoxic. II. The 78.1% of patients received at least one nephrotoxic drug. III. Some patients with decreasing GFR received more than one nephrotoxic drug [102]. In addition, the major population of participants is male, not female which the subject may make bias in investigations related to gender difference. Also, women population in clinical trials are often postmenopausal. Therefore, the lack of normal steroid hormone levels is a challenging subject in clinical trials related to gender difference. Subsequently, all of the limitations could impress the outputs of literature reviews in association with gender difference.

## 6. Conclusion

CP induces nephrotoxicity in both clinical subjects and animal models under CP therapy, and the intensity of nephrotoxicity is related to gender/sex. In addition, the effects of supplementations for preventing or reducing nephrotoxicity vary between two genders/sexes. This difference is more prominent in experiment than clinic. In other words, experimental findings are more variable than clinical findings probably due to factors such as strain, type, female estrous cycle, various experimental protocols, etc., in animal models. It is not easy to interpret this discrepancy from bench to bed. Therefore, the best strategy must choose to ameliorate or inhibit CP-induced nephrotoxicity in preclinical studies, especially in interactions between sex hormones and CP when protectors are administered. Maybe, new treatment strategies can improve CP clearance in animals or subjects receiving CP, particularly female gender/sex. Finally, there is a hopeful view, and it is necessary to perform further investigations. Up to now, the most studies related to the subjects and the findings are listed in Table 1.

## Figures and Tables

**Table 1:** The experimental and clinical studies performed in association with CP-induced nephrotoxicity.

*I. Experimental studies*				
Animal model	Cisplatin administration	Supplements	Principle findings	Ref
Male and female rats	1mg/kg/day; i.p for 14 days	—	(i) Increase of BUN, Cr, KW, and KTDS in the male more than female.	[11]
*Male and female rats*	a. Single dose of 8 mg/kg; i.p.	—	a. Increase of Cr and osmolality in both sexes. Increase of KTDS, KW, and % change of BW in the males more than females.	[7]
	b. Continuous dose of 2 mg/kg/day; i.p. for 5 days		b. Increase of Cr, osmolality, KW, KTDS, and % change of BW in both sexes.	
Male and female rats	Single dose of 7.5 mg/kg; i.p	—	(i) Increase of BUN and Cr as well as decrease of Cr clearance in 16 and 20 weeks old males more than 10 weeks old male, but not in females.	[35]
			(ii) Increase of KTDS in 16 weeks old male more than 10 and 20 weeks old males, but not in females.	
			(iii) Increase of BUN and Cr as well as decrease of Cr clearance in 10 weeks old female more than 10 weeks old male.	
Male and female monkeys	2.5 mg/kg; i.v. every 3 weeks	—	(i) Increase of BUN and Cr in the female more than male.	[26]
Male and female rats	Single dose of 0.5 mg/kg; i.v.	—	(i) Increase of renal clearance of CP in the male more than female.	[18]
			(ii) Increase of the expression of renal rOCT2 in male.	
Male and female wild type mice	Single dose of 15 mg/kg; i.p.	—	(i) Increase of Cr and KTDS and decrease of platinum accumulation in the male more than female.	[17]
*Male and female rats*	a. Single dose of 2.5 mg/kg; i.v.	—	a. Increase of 24 h urinary sodium excretion in the male treated by CP more than female.	[12]
	b. Continuous dose of 2.5 mg/kg/day; i.v. for 3 days			
			b. Increase of 24 h urinary sodium excretion in the male treated by CP, but not in female.	
Male and female mice (old and young)	Single dose of 20 mg/kg; i.p.	—	(i) Increase of Cr in the young male more than young female.	[10]
			(ii) Increase of Cr in both old male and female more than young male and female.	
			(iii) Increase of mortality rate in old female more than others.	
			(iv) Increase of KIM1 in the males more than females.	
			(v) Increase of renal MATE2 expression in the males more than females.	
Male andfemale rats	Single doses of 0.1, 1 or 2.5 mg/kg; i.p.	—	(i) Increase of urinary concentration of Cr and urinary excretion volume in the male more than female.	[15]
Male and female rats	a.Single dose of 7.5 mg/kg; i.p.	—	a and b. Increase of Cr and BUN in the female more than male.	[13]
	b. Continuous dose of 3 mg/kg/day; i.p. for 5 days			
			a. Increase of KW in the female more than male.	
			b. Increase of sodium excretion only in the female.	
Male &female mice in both type of C57 and 129/SV	Single dose of 30 mg/kg; i.p.	—	(i) Increase of BUN and Cr in the female more than male.	[25]
			(ii) No mortality in the male.	
			(iii) Increase of KTDS in the female more than male.	
Male &female mice	a. Single doses of 10 and 20 mg/kg; i.p. in order to induce AKI	—	a. Increase of mortality rate in the animals treated by the single dose of 20 mg/kg. Increase of renal damage induced by the single dose of 10 mg/kg in the male more than female.	[16]
	b. Single dose of 10 mg/kg; i.p. followed by normal diet regimen shifted to phosphate 2% diet regimen, 2 weeks after CP administration for inducing CKD			
			b. Rapid decrease of renal function in the male	
Male and female rats	Single dose of 7 mg/kg; i.p.	Single dose of L-arginine (300 mg/kg); i.p.	(i) Decrease of BUN and Cr in the male treated by L-arginine + CP	[68]
			(ii) Increase of KW and KTDS in the female treated by L-arginine + CP	
Male and female rats	Single dose of 7 mg/kg; i.p.	a.EPO (100 IU/kg/day); i.p. for 3 days as prophylaxis	(i) Decrease of BUN, Cr and MDA in the male treated by EPO + CP in both protocols (a&b), but not in female.	[83]
		b. EPO (100 IU/kg/day); i.p. as treatment		
Ovariectomized female rat	Single dose of 7 mg/kg; i.p.	ES (2.5 mg/kg/week; i.m.) for 2 weeks accompanied with Vit. E (1 g/kg/day; i.p.) or Vit. C (250 mg/kg/day; i.p.) or losartan(10 mg/kg/day; i.p.) for 7 days	(i) Increase of mortality rate in the animals treated by ES + CP accompanied by losartan or vit. C.	[8]
			(ii) Increase of BUN, Cr, KW, and KTDS in the animals treated by ES + CP accompanied with vit. E or C or losartan.	
Male and female rats	3 mg/kg/day; i.p. for 7 days	L-NAME(4 mg/kg/day; i.p.) for 7 days	(i) Increase of BUN, Cr and KTDS in the male treated by CP + L-NAME, but not in female.	[70]
			(ii) No ameliorating in BW loss in the male and female treated by CP + L-NAME.	
Male and female rats	Single dose of 7 mg/kg; i.p.	NAC at the doses of 300 and 600 mg/kg/day; i.p. for 7 days	(i) No changing in the serum levels of BUN, Cr, MDA, nitrite as well as KW and KTDS in both genders treated by CP + NAC	[73]
Male and female rats	2.5 mg/kg/day; i.p. for 7 days	L-arginine (150 mg/kg/day; i.p.) as a. Prophylaxis for 3 days	a and b. Without improvement in both sexes.	[69]
		b. Prophylaxis and treatment for 10 days	c. Decrease of BUN, Cr and KTDS in the male treated by L-arginine, but not in female.	
		c. Treatment for 7 days		
Orchiectomized male rat	2.5 mg/kg/day; i.p. for 7 days	TS at the doses of 10, 50 and 100 mg/kg/week; i.m. for 4 weeks	(i) Decrease of BUN, Cr and KTDS in the animals treated by TS 10 mg/kg/week + CP.	[33]
Ovariectomized female rat	Single dose of 7 mg/kg; i.p.	ES (500 *μ*g/kg/week; i.m.) for 4 weeks accompanied with EPO (100 IU/kg/day; i.p.) for 7 days	(i) Decrease of BUN and Cr in the animals treated by CP + EPO either with or without ES.	[80]
			(ii) Increase of nitrite as well as no attenuating KW and KTDS in the animals treated by CP + EPO accompanied with ES.	
			(iii) The improvement of KW and KTDS in the animals treated by CP + EPO without ES.	
Male and female rats	2.5 mg/kg/day; i.p. for 7 days	GABA (50 *μ*g/kg/day; i.p. for 14 days	(i) Increase of BUN, Cr and KTDS in the female treated by GABA + CP, but not in male.	[85]
			(ii) Without any improvement in the male treated by GABA + CP	
Female rat	2.5 mg/kg/day; i.p. for 7 days	PFE at the doses of 25 and 50 mg/kg/day; i.p. for 9 days	(i) No improvement in KW, BUN, Cr, and KTDS in the animals treated by PFE (25 and 50 mg/kg).	[76]
Male and female rats	Single dose of 7 mg/kg; i.p.	Losartan(10 mg/kg/day; i.p) for 9 days	(i) Increase of BUN and Cr in the females treated by CP + losartan, but not in males.	[60]
			(ii) Ameliorating BW loss and KW in the male treated by CP + losartan, but not in females	
Ovariectomized female rat	Single dose of 7 mg/kg; i.p.	ES at the doses of 0.5, 2.5 and 10 mg/kg/week; i.m. for 3 weeks	(i) No ameliorating in BUN, Cr, KW, KTDS, and BW loss in the animals treated by ES + CP.	[29]
Male &female rats	2.5 mg/kg/day; i.p. for 7 days	Enalapril (30 mg/kg/day; i.p.) for 7 days	(i) No attenuating in BUN, Cr, KTDS, KW, and BW loss in the males treated by CP + Enalapril.	[63]
			(ii) Increase of BUN, Cr, KTDS, KW, and BW loss in the females treated by CP + enalapril.	
Male and female rats	2.5 mg/kg/day; i.p. for 7 days	BOS(30 mg/kg/day) for 7 days	(i) Increase of BUN and BW loss in the males treated by BOS + CP.	[89]
			(ii) No attenuating in Cr, KW and KTDS in the males treated by BOS + CP.	
			(iii) Increase of BUN, Cr, KW and KTDS in the females treated by BOS + CP.	
			(iv) Increase of KW and KTDS in the females treated by BOS + CP than males.	
Male and female mice	2 mg/kg/day; i.p. for 3 days, then the infusion of CP was stopped for one week, again the same dose of CP was injected for 3 days, then the infusion of CP was stopped for one week, again the same dose of CP was injected for 3 days.	Riboflavin at the doses of 1 and 2 mg/kg; i.p; 30 min after CP administration and similar to CP protocol	(i) Decrease of serum urea and renal MDA in the females treated by CP + riboflavin than males.	[9]
			(ii) Improvement in the renal activities of CAT and SOD in the females treated by CP + riboflavin than males.	
Male and female rats	Single dose of 7.5 mg/kg; i.p.	Ang 1–7 (30 *μ*g/kg/day; i.p.) for 7 days	(i) Decrease of BUN, Cr, KW and KTDS in the male treated by CP + Ang 1–7, but not in female.	[64]
Male and female rats	Single dose of 7.5 mg/kg; i.p.	D 2%, 10% and 20%	(i) No survival in all males and females treated by CP + D20%	[92]
			(ii) The existence of mortality rate and no attenuating in KW in the males and females treated by CP + D2% (or D10%)	
			(iii) Increase of BUN, Cr, and KW in the females treated by CP + D10%.	
Diabetic male and female rats	2.5 mg/kg/day; i.p. for 6 days	a. GABA (50 *μ*mol/kg/day; i.p.) for 12 days as prophylaxis and treatment	(i) No changing in BUN, Cr, KW, and BW loss in both male and female treated by CP.	[88]
		b. GABA (50 *μ*mol/kg/day; i.p) for 6 days as treatment	(ii) Increase of Cr in the males treated by protocols of b&c + CP.	
		c. GABA (50 *μ*mol/kg/day; i.p) for 6 days as prophylaxis	(iii) Increase of BW loss in the males treated by three protocols + CP.	
			(iv) No changing in BUN, Cr, KW and BW loss in the females treated by three protocols + CP.	
Female rat	2.5 mg/kg/day; i.p. for 7 days	Aerobic exercise for eight weeks	(i) No ameliorating in BUN, Cr, KW, and KTDS in the animals treated by EX + CP.	[97]
			(ii) Increase of KTDS in the animals treated by EX + CP.	
Male and female rats	Single dose of 7.5 mg/kg; i.p.	Five protocols of hydration: a. Saline	(i) More survival rate in the males than females	[94]
		b. Mannitol	(ii) Increase of mortality rate in the female treated by protocols of b&c.	
		c. Saline-D	(iii) Increase of mortality rate, BUN, Cr, and sodium excretion fraction as well as decrease of Cr clearance in the male treated by mannitol.	
		d. Saline-furosemide	(iv) Increase of BUN, Cr, and sodium excretion fraction and decrease of Cr clearance in the female treated by protocols of d&e.	
		e. Saline-mannitol		
Male and female rats	Single dose of 7 mg/kg; i.p.	EPO (100 IU/kg/day, i.p) or Vit. E (250 mg/kg/day; i.p) or NAC (600 mg/kg/day; i.p.) for 7 days	(i) Decrease of serum NOX and nitrite levels in the males treated by CP + vit.E, but not in female.	[82]
			(ii) No attenuating the serum levels of NOX and nitrite in the male and female treated by EPO or NAC.	
Male and female rats	Single dose of 7 mg/kg; i.p.	Vit. E (1 g/kg/day; i.p.) for 7 days	(i) Increase of BUN and Cr and KTDS in the female treated by CP + vit.E.	[81]
			(ii) The improvement of BUN, Cr, and KTDS in the male treated by CP + Vit.E	
Male and female rats	2.5 mg/kg/day; i.p for 6 days	SMT (5 mg/kg, two times/day; i.p.), for 6 days	(i) Decrease of BUN, Cr, nitrite, and MDA in the male treated by SMT + CP.	[71]
			(ii) Decrease of Cr and increase of KW in the female treated by SMT + CP.	
Ovariechtomized female rat	2.5 mg/kg/day; i.p. for 8 days	a. Pro at the doses of 2, 5, 10, and 25 mg/kg; i.m. every 5 days for 3 cycles	a. Increase of Cr in the animals treated by CP + Pro 2 or Pro5.	[31]
		b. The combination of Pro (10 mg/kg) with ES at the doses of 0.25, 0.5 and 1 mg/kg; i.m. every 5 days for 3 cycles	(ii) Increase of kidney MDA in the animals treated by CP + Pro25.	
			(iii) Decrease of KW, KTDS, BW change, and nitrite in the animals treated by CP + Pro10. b. Decrease of Cr and BUN in the animals treated by CP + ES/Pro	
			(iv) Increase of KW in the animals treated by CP + Pro 10/Es 0.5 or 1.	
			(v) Decrease of KTDS in the animals treated by CP + Pro 10/ES 0.25 or ES 0.5.	
			(vi) Decrease of serum MDA in all animals treated by CP + Pro/ES.	
			(vii) Increase of serum nitrite in the animals treated by CP + Pro 10/ES 0.5.	
			(ix) Increase of renal MDA in the animals treated by CP + Pro 10/ES 0.5 or ES 1.	
*Gonadectomized male and female rats*	a. Continuous dose of 3 mg/kg/day; i.p for 5 days	TS 10 mg/kg/week; i.m. for 3 weeks or ES 0.25 mg/kg/week; i.m. for 3 weeks	a. Increase of BW loss and no attenuating in KTDS and KW in the animals treated by TS.	[30]
	b. The single dose of 7.5 mg/kg; i.p.		(i) No attenuating in KW, KTDS, attenuating BW loss in the animals treated by ES. b. Attenuating BUN and Cr and BW loss, KTDS in the animals treated by ES.	
			(ii) No attenuating in KW in the animals treated by ES.	
*II. Clinical studies*				
Subject	Chemotherapy protocol	Supplements	Principle findings	Reference
Men and women	Not available	Not available	(i) Increase of AKI in men than women.	[58]
Men and women	Not available	Not available	(i) Increase of nephrotoxicity in the premenopausal women than men.	[47]
Old men and women	CP (60–75 mg/m2) or carboplatin accompanied with pemetrexed every 3 weeks	—	(i) No association between gender and nephrotoxicity.	[44]
Old men and women	CP at the mean dose of 27 mg/m2/week	—	(i) No relations between serum Cr, Cr clearance, and gender.	[42]
Men and women	CP at the blues dose of 100 mg/m2 accompanied with 5- Fluorouracil and radiotherapy	Aggressive hydration with saline, KCl, mannitol, MgSO_4_, and D5	(i) No association between gender and nephrotoxicity.	[43]
Men &women	CP accompanied with radiotherapy: a.CP at the dose of 100 mg/m2 once every three weeks in order to AKI	Hydration with normal saline, mannitol, KCl, and MgSO_4_	a. Increase of AKI in women more than men.	[50]
	b. CP at the average dose of 225.6 mg/m2 in order to chronic kidney injury		b. Decrease of eGFR in the women more than men.	
Men and women	CP at the mean dose of 84 mg/m2 every three weeks in the combination with other chemotherapy drugs	Hydration with glucose 5% containing calcium glucoronate, magnesium pyrrolidone carboxylate, KCl, NaCl	(i) No correlation between gender and renal dysfunction.	[38]
Men and women	CP at the dose of 75–80 mg/m2 every three weeks in the combination with other chemotherapy drugs	Hydration with saline and MgSO_4_	(i) No correlation between gender and nephrotoxicity.	[41]
Men and women	CP at the dose of 80 mg/m2 in combination with other chemotherapy drugs	Hydration with mannitol, normal saline, hypotonic crystalloid solution, and furosemide	(i) Shorter interval between the start of chemotherapy and the development of serum Cr elevation in the women than men.	[52]
Men and women	CP at the dose of 70–85 mg/m2 alone or CP at the dose of 70 mg/m2 in the combination with other chemotherapy drugs	Hydration with normal saline, D-saline, KCl, and MgSO_4_	(i) Two times risk factor of nephrotoxicity in the women more than men.	[2]
Men and women	CP dose was not available	Short hydration with magnesium supplement	(i) Female gender was risk factor in CP-induced renal damage.	[48]
Men and women	CP at the dose of 50–99 mg/m2 alone or in combination with other chemotherapy drugs	Aminophylline at the doses of 4 and 0.4 mg/kg; i.v before and after CP administration, respectively as well as the oral administration of theophylline tablets at the dose of 200 mg; three times/day for four days. Hydration with isotonic saline, KCl, and MgSO_4_	(i) No changing in CP-induced nephrotoxicity in the patients treated with aminophylline and theophylline.	[102]
			(ii) No correlation between gender and CP-induced nephrotoxicity.	
Men and women	CP at the dose of 175–225 mg/m2 alone as intraoperative intracavitary lavage	Hydration with normal saline.	(i) Increase of AKI in the men more than women.	[99]
		Amifostine		
Men &women	CP at the dose of 50–100 mg/m2 alone or in the combination with other chemotherapy drugs every 21 days	Hydration with isotonic saline, KCl, and MgSO_4_	(i) Decrease of CP-induced nephrotoxicity in the patients treated with hydration.	[53]
			(ii) The number of women patients with CP-induced nephrotoxicity less than men.	
Men and women	CP at the dose of 80 mg/m2 in the combination with other chemotherapy drugs	Hydration with mannitol	(i) Increase of Cr during first cycle of CP, regardless gender	[57]
			(ii) Increase of nephrotoxicity intensity in the men treated by generic CP than those treated by brand name CP, but not in women.	
Men and women	CP at the dose of below 100 mg/m2 in the combination with radiotherapy	Hydration with mannitol and diuretic	(i) No association between gender and renal toxicity.	[40]
Men and women	CP alone at the cumulative dose of ≤100 and >701 mg/m2	Hydration with normal saline	(i) Decrease of eGFR in the women.	[49]

AKI: acute kidney injury; Ang 1–7: angiotensin 1–7; BOS: bosentan; BUN: blood urea nitrogen; BW: body weight; CAT: catalase; CKD: chronic kidney disease; CP: cisplatin; Cr: creatinine; D: dextrose; eGFR: estimated glomerular filtration rate; EPO: erythropoietin; ES: estradiol valerate; EX: exercise; GABA: gamma-aminobutyric acid; i.m: intramuscularly; i.p.: intraperitoneally; i.v.: intravenously; KCl: potassium chloride; KIM1: kidney injury molecule 1; KTDS: kidney tissue damage score; KW: kidney weight; L-NAME: N(*ω*)-nitro-L-arginine methyl ester; MATE1: multidrug and toxin extrusion1; MDA: malondialdehyde; mGFR: measured glomerular filtration rate; MgSO_4_: magnesium sulphate; NAC: N-acetyl cysteine; PFE: pomegranate flower extract; Pro: progesterone; rOCT2: rat organic cation transporter2; SMT: S-methylisothiourea sulphate; SOD: superoxide dismutase; TS: testosterone; Vit: vitamine.

## Data Availability

The content material is available by request.
